# 1245. *In Vitro* Activities of Ceftaroline and Comparator Agents Against Bacterial Pathogens Collected from Patients with Skin and Skin Structure Infections: ATLAS Global Surveillance Program 2012-2019

**DOI:** 10.1093/ofid/ofab466.1437

**Published:** 2021-12-04

**Authors:** Meredith Hackel, Gregory Stone, Daniel F Sahm

**Affiliations:** 1 IHMA, Inc., Schaumburg, Illinois; 2 Pfizer, Inc., Groton, CT

## Abstract

**Background:**

Ceftaroline fosamil, the prodrug of ceftaroline, is a parenteral cephem approved for the treatment of patients with skin and skin structure infections (SSSIs) caused by *Staphylococcus aureus* (both methicillin-susceptible [MSSA] and methicillin-resistant [MRSA] isolates), β-hemolytic streptococci (*Streptococcus pyogenes, S. agalactiae, S. dysgalactiae*), and select species of Enterobacterales (*Escherichia coli, Klebsiella pneumoniae, Klebsiella oxytoca*). The current study is part of the ATLAS (Antimicrobial Testing Leadership and Surveillance) program and evaluated the current activities of ceftaroline and comparator agents against commonly encountered bacterial isolates associated with SSSIs.

**Methods:**

From 2012 to 2019 the ATLAS program received 124,694 bacterial isolates that had been cultured by 493 clinical laboratories in 71 countries from samples of patients diagnosed with SSSIs. All isolates were transported to IHMA, (Schaumburg, IL, USA) where their identities were confirmed using MALDI-TOF mass spectrometry and antimicrobial susceptibility testing performed following standardized CLSI broth microdilution methodology (M07). Percent susceptibilities were determined using 2021 CLSI MIC breakpoints. Phenotypic extended-spectrum β-lactamase (ESBL) screening and confirmatory testing were performed using the CLSI M100 method.

**Results:**

The *in vitro* activity of ceftaroline is summarized in the following table. Overall, >99.9% of MSSA and 92.8% of MRSA from SSSI were susceptible to ceftaroline (MIC ≤1 µg/ml); 7.1% of MRSA isolates were ceftaroline-susceptible dose-dependent (MIC 2-4 µg/ml) with greatest proportion being from Chile (53.3% of 392 isolates), S. Korea (29.3% of 321 isolates), and China (24.7% of 652 isolates). Twelve ceftaroline-resistant MRSA were observed, consisting of 11 of 109 isolates from Thailand (10.1%) and 1 of 161 from China (0.6%). All *S. pyogenes* and 88.0% of ESBL-negative Enterobacterales were susceptible to ceftaroline.

Results Table

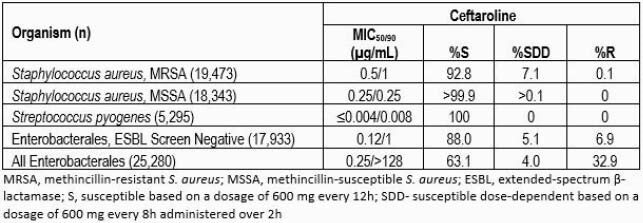

**Conclusion:**

Ceftaroline continues to demonstrate potent *in vitro* activity against clinically relevant pathogens associated with SSSIs.

**Disclosures:**

**Meredith Hackel, PhD MPH**, **IHMA** (Employee)**Pfizer, Inc.** (Independent Contractor) **Gregory Stone, PhD**, **AztraZeneca** (Shareholder, Former Employee)**Pfizer, Inc.** (Employee) **Daniel F. Sahm, PhD**, **IHMA** (Employee)**Pfizer, Inc.** (Independent Contractor)

